# Tissue harvest with a laser microbiopsy

**DOI:** 10.1117/1.JBO.27.12.125001

**Published:** 2022-12-14

**Authors:** Jason B. King, Nitesh Katta, Sapun H. Parekh, Thomas E. Milner, James W. Tunnell

**Affiliations:** a The University of Texas at Austin, Department of Biomedical Engineering, Austin, Texas, United States; b University of California Irvine, Beckman Laser Institute and Medical Clinic, Irvine, California, United States

**Keywords:** laser ablation, microbiopsy, minimally invasive, virtual hematoxylin and eosin

## Abstract

**Significance:**

Traditional pathology workflow suffers from limitations including biopsy invasiveness, small fraction of large tissue samples being analyzed, and complex and time-consuming processing.

**Aim:**

We address limitations of conventional pathology workflow through development of a laser microbiopsy device for minimally invasive harvest of sub-microliter tissue volumes. Laser microbiopsy combined with rapid diagnostic methods, such as virtual hematoxylin and eosin (H&E) imaging has potential to provide rapid minimally invasive tissue diagnosis.

**Approach:**

Laser microbiopsies were harvested using an annular shaped Ho:YAG laser beam focused onto the tissue surface. As the annulus was ablated, the tissue section in the center of the annulus was ejected and collected directly onto a glass slide for analysis. Cryogen spray cooling was used before and after laser harvest to limit thermal damage. Microbiopsies were collected from porcine skin and kidney. Harvested microbiopsies were imaged with confocal microscopy and digitally false colored to provide virtual H&E images.

**Results:**

Microbiopsies were successfully harvested from porcine skin and kidney. Computational and experimental results show the benefit of cryogen pre- and post-cooling to limit thermal damage. Virtual H&E images of microbiopsies retained observable cellular features including cell nuclei.

**Conclusions:**

Laser microbiopsy with virtual H&E imaging shows promise as a potential rapid and minimally invasive tool for biopsy and diagnosis.

## 1 Introduction

Tissue harvest and examination is widely used for diagnosis and intraoperative margin assessment of disease including many types of cancer. Examination of thinly sliced and hematoxylin and eosin (H&E) stained tissue sections is the gold standard despite numerous well-known limitations. The traditional workflow involves: (1) harvest of tissue sections on the order of a mm or larger using mechanical tools such as punches, needles, scalpels, or forceps, (2) preparing the tissue for sectioning by either fixing and embedding in paraffin (>1  day) or by freezing, (3) slicing the tissue into thin sections (3 to 10  μm), (4) staining with H&E (>20  min),^1^ and (5) microscopic examination by a pathologist. Limitations of this workflow include biopsy invasiveness, small fraction of harvested tissue analyzed, relatively long time required for processing, human factors including need for highly trained technicians, and artifacts from processing and sectioning.^2^

Limitations of the traditional H&E workflow have been well recognized leading to significant research and development of alternative techniques. Non-invasive or minimally invasive sampling methods have been proposed to replace traditional biopsies. Non-invasive methods include probe devices such as mass spectrometry^3^ or optical spectroscopy^4^ probes as well as tape stripping.^5^ Minimally invasive microbiopsy tools have been designed to mechanically cut and harvest sub-microliter ($<1\;{\mathrm{mm}}^{3}$) tissue volumes.^6^^–^^9^ These microbiopsies are hundreds of microns in diameter, making them compatible with the traditional H&E workflow in addition to compatibility with alternative methods, such as mass spectrometry, optical spectroscopy, and genetic testing. We present an alternative approach using a laser for minimally invasive microbiopsy. While lasers have been utilized previously for laser microdissection to harvest cells from thin histologic sections or cell cultures,^10^^–^^14^ the laser microbiopsy approach described here harvests three-dimensional tissue volumes from thick tissues. In addition, we describe a photothermal cutting mechanism as opposed to plasma mediated ablation used for laser microdissection.^13^^,^^14^ Advantages of laser vs. mechanical tissue harvest include non-contact cutting, precise adjustable laser beam shaping, and fiber optic delivery.

Additionally, progress has been achieved on creating H&E looking images without sectioning and with minimal to no tissue processing. These “virtual H&E” images can be generated using microscopy techniques including confocal,^15^^,^^16^ two-photon,^17^^–^^19^ light-sheet,^20^ ultraviolet surface excitation,^21^ structured illumination,^22^ optical coherence tomography (OCT),^23^ and stimulated Raman scattering.^24^^,^^25^ Virtual H&E images can be generated within minutes of tissue harvest, do not require a histology technician to process the tissue, and do not have artifacts that result from traditional processing and sectioning.

We propose a workflow for rapid, minimally invasive tissue harvest and analysis. An annular shaped laser beam is used to cut sub-microliter tissue volumes. The tissue volumes are ejected from the bulk tissue and are collected directly onto a glass slide for analysis. Cryogen spray cooling is used before and after tissue harvest to reduce tissue damage. Harvested tissue sections are rapidly stained, imaged with confocal microscopy, and false colored to create virtual H&E images within minutes of harvest.

## 2 Materials and Methods

### 2.1 Laser Microbiopsy Overview

**Fig. 1:**
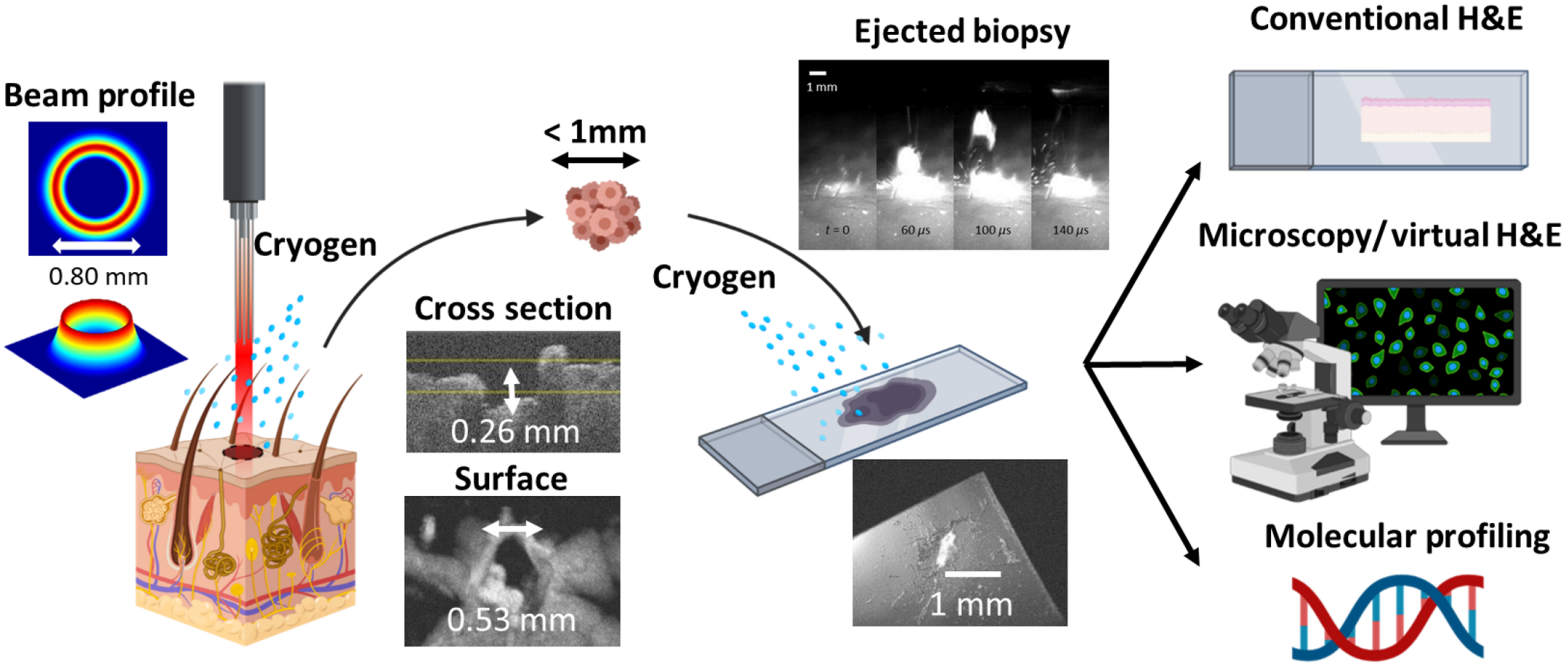
Overview of laser microbiopsy workflow. An annular beam is used to cut a sub-microliter tissue section. Cryogen spray cooling is used pre- and post-ablation to limit thermal damage. The tissue is ejected and collected directly onto a glass slide for imaging/analysis. Figure and portions of methods adapted from Refs. 26 and 27.

An overview of the laser microbiopsy approach is shown in Fig. 1. A pulsed laser beam is shaped through a lens system to generate a converging annular beam profile at the tissue surface. The annular beam shape ablates the tissue, and the tissue section in the center of the annulus is ejected. Cryogen spray cooling is used before and after the laser pulse to limit tissue damage and patient discomfort. These tissue sections, with volumes <1  μl ($=1\;{\mathrm{mm}}^{3}$), are collected onto a glass slide where they can be analyzed using desired methods including, but not limited to, conventional H&E staining, virtual H&E or other microscopy methods, and molecular profiling.

### 2.2 Benchtop Laser Microbiopsy Implementation

**Fig. 2:**
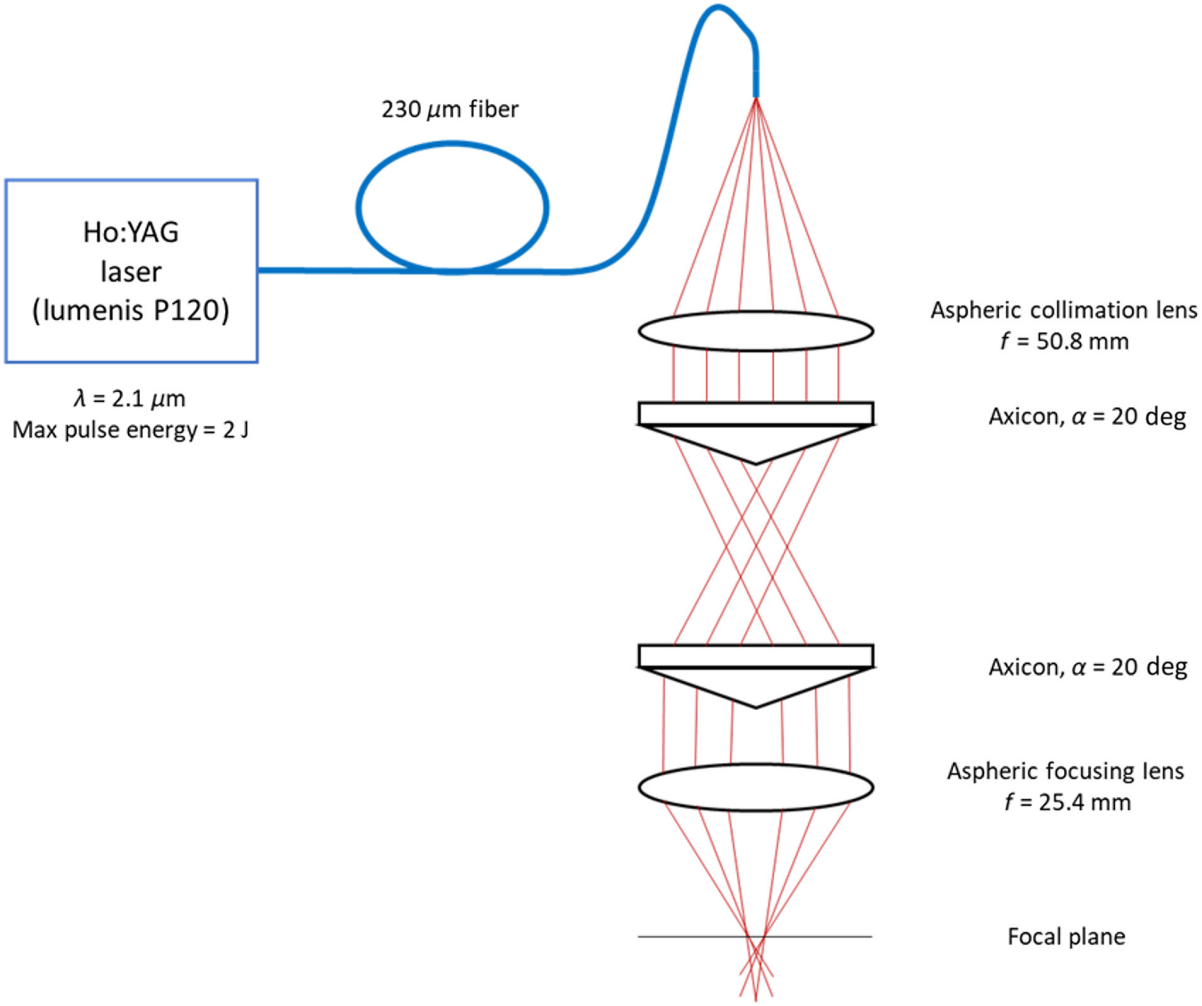
Diagram of optics used in this study. A Ho:YAG laser is shaped into an annular beam using a pair of axicons. The annular beam is focused on the tissue surface through an aspheric focusing lens. Cooling and harvesting slide not shown.

We have designed and built a benchtop laser microbiopsy system consisting of the following components (Fig. 2): (1) A Ho:YAG laser (MOSES Pulse 120H, Lumenis, Yokneam, Israel) delivered through a 230-μm core diameter multimode fiber (Moses 200 D/F/L, Lumenis, Yokneam, Israel). An SMA connector was added to the distal end of the fiber to allow connection to a fiber mount with x-y-z positioning and tilt control for precise optical alignment. (2) A ZnSe aspheric collimation lens (ASPH-ZC-25-50, ISP Optics, Orlando, Florida) to collimate light from the fiber. (3) Two fused silica axicons (α=20  deg, Thorlabs, Newton, New Jersey) to shape the collimated circular beam into an annular beam. The axicons are oriented with the points of the conical surface facing in the direction of light propagation as opposed to the typical orientation with the axicon points facing each other. This axicon orientation provides an annular beam diverging at an angle of 0.6 deg rather than perfect collimation, which results in the beam retaining an annular shape at the focal plane. (4) A ZnSe aspheric focusing lens (ASPH-ZC-25-25, ISP Optics, Orlando, Florida) is utilized to focus the annular shape. The optical components produce a converging annular shape with decreasing diameter towards the beam focus. A similar beam shaping approach has been reported for material processing using an annular beam.^28^ The converging annular shape conveniently allows a variable annulus diameter at the tissue surface by adjusting the distance between the focusing lens and the tissue surface. Additionally, the beam converges into the tissue, generating a pressure gradient necessary to eject the harvested tissue upward where it is collected directly onto a glass slide. Cryogen spray cooling (R134A) is used before and after pulsed laser emission to limit thermal damage. A solenoid fuel injector valve (800-1257N, GP Sorensen, Long Island City, New York) is used to deliver the cryogen spray pre-cooling directed at the tissue surface from 4 cm away and a second valve is used to deliver post-cooling directed at the biopsy collected on the glass slide from 4 cm away.

### 2.3 Transmission Measurement

Transmission through the beam-shaping optics was measured. A power meter (PM10, Coherent, Santa Clara, California) was used to record average power before and after the beam-shaping optics, and percent transmission was calculated.

### 2.4 Beam Profile Measurement

The annular beam profile produced by our benchtop system was explored computationally and experimentally. The optical components were imported into raytracing software (Zemax), and the resulting beam profile was measured in software with a virtual detector. The beam profile was measured experimentally by irradiating a 100-μm layer of water in a rectangular capillary tube and imaging the emitted radiation using an IR camera (420-0044-04-00, FLIR, Wilsonville, Oregon) [Fig. 5(a)]. For these measurements, the laser pulse energy was set to the minimum level (0.2 J). Pulse energy was further reduced by coupling light between two optical fibers with intentionally poor coupling efficiency. Beam-shaping optics were oriented horizontally, and the rectangular capillary was placed a variable distance, Z, from the back surface of the focusing lens. The capillary was initially placed at Z=20  mm and increased in increments of 0.25 mm to measure the beam profile at varying focal locations. A ZnSe lens (ASPH-ZC-25-50, ISP Optics, Orlando, Florida) was placed between the capillary and the IR camera to magnify the beam profile image. A video was recorded by the IR camera at a frame rate of 432.5 Hz and an integration time of 0.50 ms. The first frame after laser emission was recorded and used for beam profile analysis. Computational and experimental beam profiles were compared.

### 2.5 Computational Modeling

The laser microbiopsy processes were modeled computationally to gain insight in beam-shaping optimization, interaction with annular beam and tissue, tissue ablation, and thermal damage. The simulation is based off of a blow-off model as described by Vogel and Venugopalan.^29^ This model is conditional on the laser pulse being shorter than the thermal diffusion time providing thermal confinement. In the case of annular ablation, we calculate the thermal diffusion time using Eq. (1), where l is the width of the annular beam from inner to outer diameter, α is the thermal diffusivity, k is the thermal conductivity, ρ is the tissue density, and $C_{p}$ is the tissue specific heat capacity (Table 1)

$$t_{d}=\frac{l^{2}}{4\alpha },\;\alpha =\frac{k}{\rho C_{p}}.$$ (1)

**Table 1:** Parameters used in computational modeling.

Parameter	Value
Pulse energy	1 J
Absorption coefficient, $\mu_{a}$	$30\;{\mathrm{cm}}^{-1}$
Reduced scattering coefficient, $\mu_{s}^{\prime }$	$6\;{\mathrm{cm}}^{-1}$, $3\;{\mathrm{cm}}^{-1}$, 0
Scattering phase function	Henyey–Greenstein^30^
Anisotropy factor, g	0.9
Tissue density, ρ	$1109\;{\mathrm{kg}/m}^{3}$
Specific heat capacity, $C_{p}$	3391 J/kgK
Thermal conductivity, k	0.4 W/mK
Convective heat transfer coefficient, h	$10\;W/m^{2}\;K$, $10,000\;W/m^{2}\;K$
External convective medium temperature, $T_{\mathrm{ext}}$	23°C, −40°C^31^
Arrhenius frequency factor, A	$4.585e72\;s^{-1}$ ^32^
Arrhenius activation energy, $E_{a}$	4.710e5 J/mol^32^

Using an annular beam width of 100  μm gives a thermal diffusion time of 24 ms. The pulse duration of the laser is much shorter with a FWHM duration of 71  μs.^33^ Therefore, thermal confinement during the laser pulse is a justified assumption, and the blow-off model can be used.

**Fig. 3:**
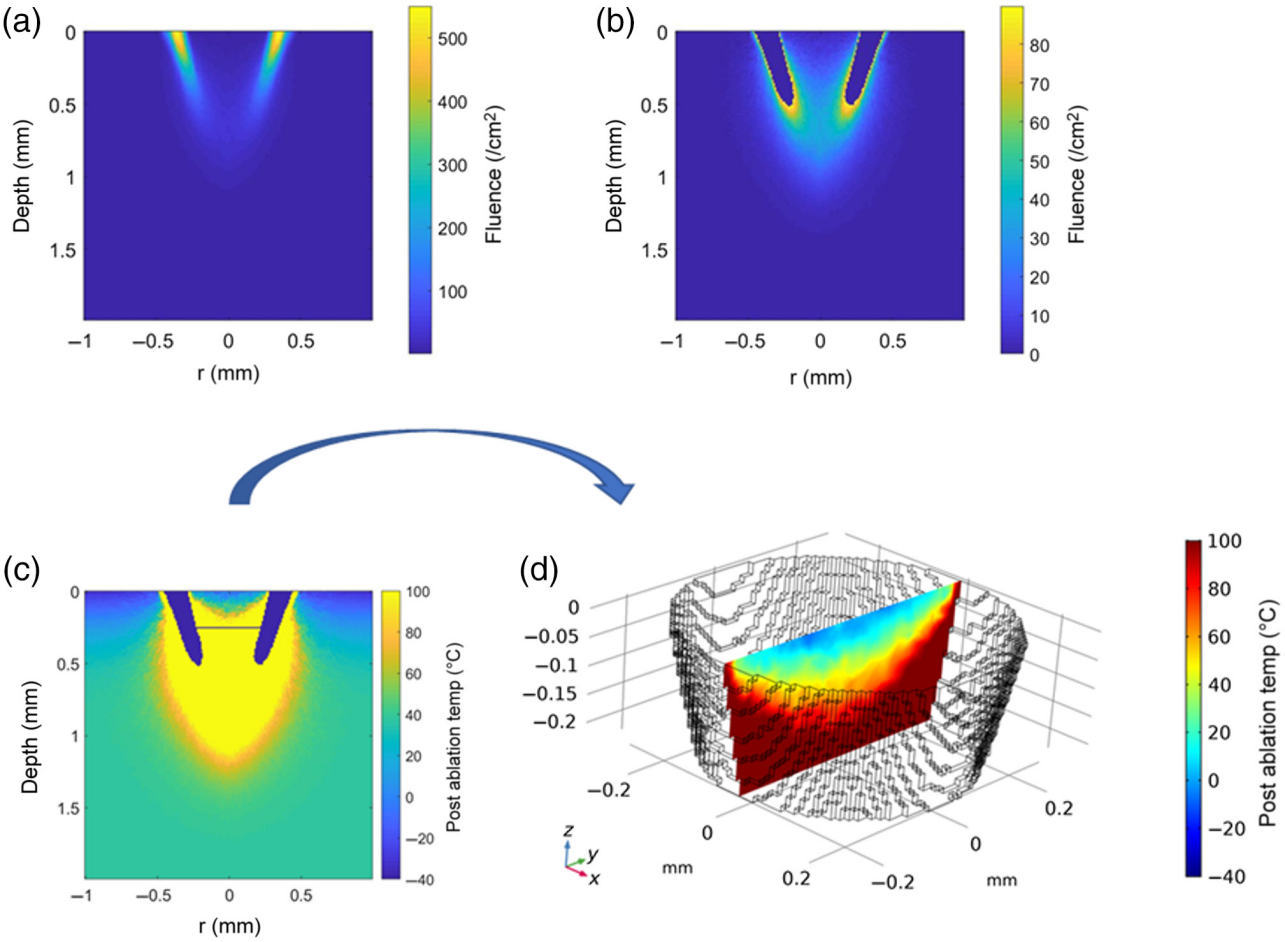
Computational model steps. (a) Fluence through the tissue was modeled using Zemax. (b) Regions with fluence above the ablation threshold were removed [displayed as dark blue in (b) and (c)]. (c) The microbiopsy separated from the bulk tissue at the conclusion of the laser pulse and the post ablation temperature evolution was calculated. (d) The microbiopsy volume and initial temperature were exported to COMSOL for modeling of bioheat transfer and thermal damage.

The computational model consisted of the following steps. (1) The optical fiber and beam-shaping optics were modeled in raytracing software (Zemax), and the beam profile at varying distance from the beam focus was simulated. (2) A volume detector with the absorption and scattering properties of soft tissue (Table 1) was inserted at a distance such that the outer diameter of the annular beam was 800  μm. (3) The simulated fluence in the tissue was computed with raytracing and exported to MATLAB [Fig. 3(a)]. 4) Regions with a radiant energy density greater than a threshold of $2.5\;J/{\mathrm{mm}}^{3}$ were assumed to be ablated, and the tissue inside the ablation annulus was assumed to be ejected as a microbiopsy at the conclusion of the laser pulse [Fig. 3(b) and 3(c)]. The ablation threshold is derived from the energy required to vaporize water within the tissue.^34^ Energy absorbed in the ablation region was removed from the model as this energy is assumed to be spent on ablation and kinetic energy of tissue expulsion. The energy absorbed in the regions of tissue that are not ablated contributes to tissue heating. The temperature at the end of the laser pulse at each depth and radius, is calculated using Eq. (2), where 37°C is the initial body temperature, F(r,z) is the fluence at each location, $\mu_{a}$ is the absorption coefficient, ρ is the tissue density, and $C_{p}$ is the tissue specific heat capacity (Table 1):^29^

$$T(r,z)=37{}^{\circ}C+\frac{F(r,z)\mu_{a}}{\rho C_{p}}.$$ (2)

(5) The simulated biopsy was severed at half of the maximum ablation depth to give a simulated microbiopsy volume within the range of experimental microbiopsy volumes. The volume and geometry of the tissue in the middle of the ablated annulus and above the severed depth were saved to an STL file and imported into COMSOL multiphysics (COMSOL) as the volume of the ejected microbiopsy [Fig. 3(d)]. The temperature at the end of the laser pulse [Eq. (2)] within the ejected microbiopsy volume was imported into COMSOL [Figs 3(c) and 3(d)]. (6) The heat transfer through the tissue as it cools was modeled in COMSOL. (7) The Arrhenius damage integral [Eq. (3)] and the percentage of cell death [Eq. (4)] were calculated in COMSOL for each location in the microbiopsy.^29^^,^^35^^,^^36^

In these equations, Ω is the Arrhenius thermal damage parameter, A is the frequency factor, $E_{a}$ is the activation energy per mole, R is the universal gas constant, and T is the tissue temperature in Kelvin. Values used for these parameters are given in Table 1. Importantly, the Arrhenius damage integral is used here as an overall measure of thermal damage to make comparisons for optimization but does not necessarily indicate whether the tissue will retain integrity for analysis by virtual H&E or other analysis methods. An Arrhenius thermal damage parameter of Ω=1 indicates 63% cell death and is used as a threshold for thermal damage.

$$\Omega (\tau )=\int_{0}^{\tau }A*e^{\left(\frac{-E_{a}}{R*T(t)}\right)}dt,$$ (3)

$$\text{Damage }(\%)=100*(1-e^{-\Omega }).$$ (4)

This model allows for various parameters to be varied to better understand the laser microbiopsy process and aid in optimization to limit microbiopsy thermal damage. Important parameters that were explored included tissue scattering coefficient, convective heat transfer coefficient, and external temperature. The effects of cryogen spray cooling before (pre-cooling) and/or after (post-cooling) the laser pulse were explored. To model pre-cooling, the heat transfer through tissue was calculated with a surface temperature of −40°C and a convective heat transfer coefficient at the surface of $10,000\;W/m^{2}\;K$ for a duration of 200 ms. Detailed descriptions of cryogen spray cooling including computational models and experimental results can be found elsewhere.^31^^,^^37^^–^^39^ To model post-cooling the convective heat transfer coefficient and external temperature were set to $10,000\;W/m^{2}\;K$ and −40°C. To model cooling in room air, the convective heat transfer coefficient and external temperature were set to $10\;W/m^{2}\;K$ and 23°C.

The effects of tissue scattering were also explored. The model was run with normal tissue reduced scattering coefficient (${\mu_{s}}^{\prime }=6\;{\mathrm{cm}}^{-1}$),^40^ scattering reduced by half (${\mu_{s}}^{\prime }=3\;{\mathrm{cm}}^{-1}$), and without scattering (${\mu_{s}}^{\prime }=0$). Experimental results with reduced tissue scattering are not available for microbiopsy volume comparison. Therefore, biopsy volumes were assumed to separate from the bulk tissue at the maximum ablation depth for these models.

**Fig. 4:**
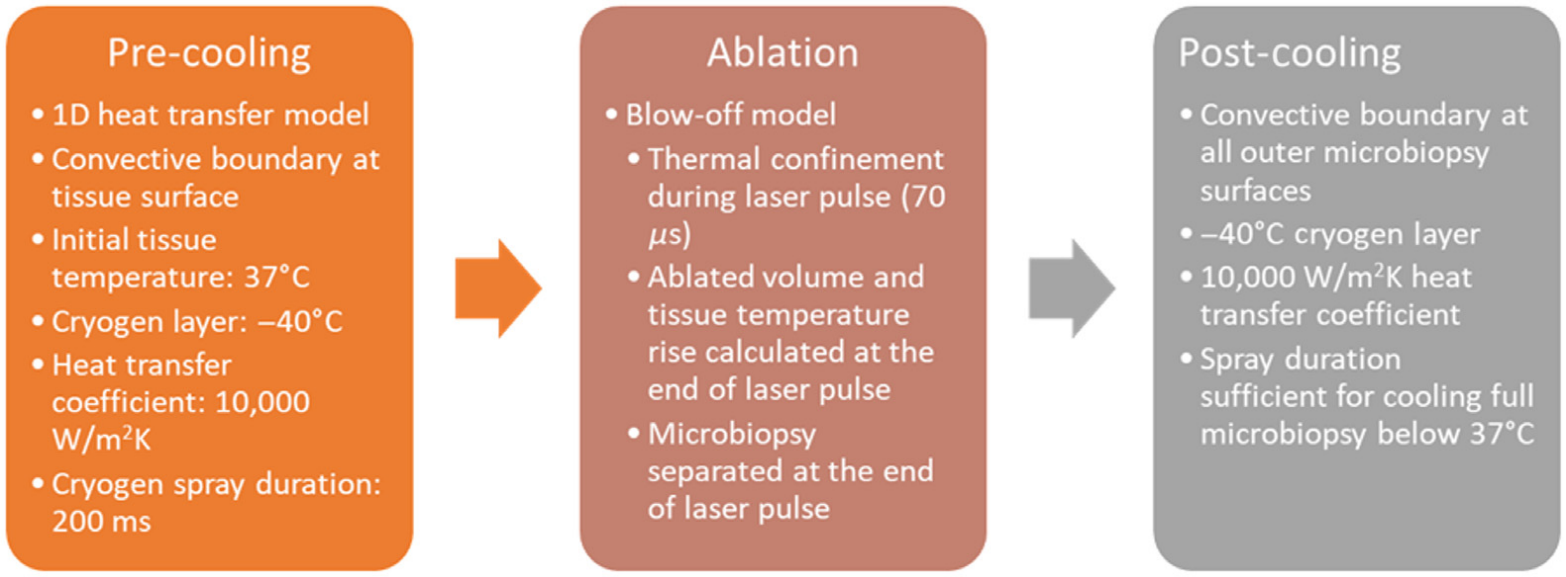
Key assumptions made in the thermal damage model for each stage: pre-cooling, ablation, and post-cooling.

The full set of parameters used in the computational model is summarized in Table 1. The key assumptions made in this model are summarized in Fig. 4.

### 2.6 Microbiopsy Harvest Experiments

Microbiopsy harvest experiments were completed on *ex vivo* porcine tissues (Animal Technologies, Tyler, Texas). The protocol involved the following: (1) the tissue surface was aligned to the desired position relative to the beam focus using an aiming beam. This position determines the diameter of the annular beam at the tissue surface. (2) A 100-μm thick glass coverslip was positioned ∼5  mm above the tissue surface to collect the ejected microbiopsy. (3) A custom shutter consisting of a foil paddle connected to a rotational stepper motor (17md302s-00, Anaheim Automation, Anaheim, California) was placed with the foil paddle initially directly below the focusing lens, preventing initial pulses emitted by the laser from reaching the tissue. (4) Pre- and post-cooling solenoid valves were directed to the tissue surface and glass coverslip, respectively. (5) The laser pulse energy was set. (6) A train of pulses was manually initiated from the laser. The first pulse emitted by the laser was detected by an InGaAs photodetector (2034, New Focus, San Jose, California) connected to a digital delay generator. This event was used to control the shutter and spray cooling timing. (7) Pre-cooling began 600 ms before microbiopsy collection for a duration of 400 ms. (8) The shutter rotated open to allow a single laser pulse to perform the microbiopsy before returning to the closed position to block the following pulse. (9) Post-cooling began immediately after microbiopsy collection for a duration of 200 ms.

This procedure was tested on *ex vivo* porcine skin and kidney tissues. Optimal pulse energy and tissue location in the beam (beam diameter) were experimentally determined for each tissue type. A fast video camera (Fastcam Mini UX100, Photron, Tokyo, Japan) was used to record the skin ablation process at 50,000 fps.

### 2.7 Microbiopsy Volume Measurements

Microbiopsies were collected from *ex vivo* porcine skin at two distances from the focusing lens (separated by 0.5 mm) providing a larger and smaller beam with estimated inner annulus diameters of 530 and 420  μm, respectively. Beam diameters outside this range were either too large or small to reliably harvest microbiopsies. Pulse energies of 1 and 1.3 J incident on the tissue surface were used. Pulse energies <1  J were insufficient to reliably harvest microbiopsies from porcine skin. Five microbiopsies were collected for each combination of beam diameter and pulse energy. Harvested microbiopsies were imaged using a custom-built OCT system.^36^ The OCT system used a single mode optical fiber with a swept source laser (1310 nm, Axsun, Billerica, Massachusetts). The OCT system used a Mach–Zehnder fiber interferometer configuration collecting field of views up to 6.5  mm(X)×6.5  mm(Y)×3  mm (depth). A custom MATLAB program was used to detect the microbiopsy surface from recorded OCT data, display a 3D reconstruction of each microbiopsy, and calculate microbiopsy volumes.

### 2.8 Virtual H&E

To generate H&E images, acridine orange (AO) was chosen as the nuclear stain with sulforhodamine 101 (SR101) as a counter stain as described by Cahill et al.^17^ The stain was prepared by mixing 8 mg AO (A6014, Sigma-Aldrich, St. Louis, Missouri) and 5 mg SR101 (HB0838, Hello Bio, Princeton, New Jersey) in 50 ml 1:1 water:ethanol. Tissues were stained for 2 min followed by a 20 s rinse in 1:1 water:ethanol. Tissues were then imaged with a confocal microscope (Ultima IV, Bruker, Billerica, Massachusetts) with a 20× objective. Two excitation wavelengths were used: 488 nm for AO excitation and 561 nm for SR101 excitation. Two emission channels were used: 525±25  nm for AO emission and 605±35  nm for SR101 emission. The two channels were false colored and merged as described by Gareau^15^ with the SR101 channel taking the place of the reflectance channel. The R and G values for the AO or H channel were increased to reduce blueness after feedback from a pathologist. The RGB values used for the H&E digital staining were H=[0.5,0.4,1] and E=[1,0.55,0.88]. Virtual H&E imaging was first done on bulk tissues as a control. Microbiopsies and ablation craters were then imaged and compared to bulk tissues to evaluate damage.

## 3 Results

### 3.1 Transmission Measurements

Transmission through the beam-shaping optics was measured to be 66% of pulsed radiant energy released from the fiber tip. Therefore, a pulse energy setting at the laser of 2 J results in a pulse energy at the tissue surface of 1.3 J while a pulse energy setting of 1.5 J results in a pulse energy at the tissue surface of 1 J. All values reported in this study are the Ho:YAG radiant pulse energy incident on the tissue surface.

### 3.2 Simulated and Experimental Beam Profiles

**Fig. 5:**
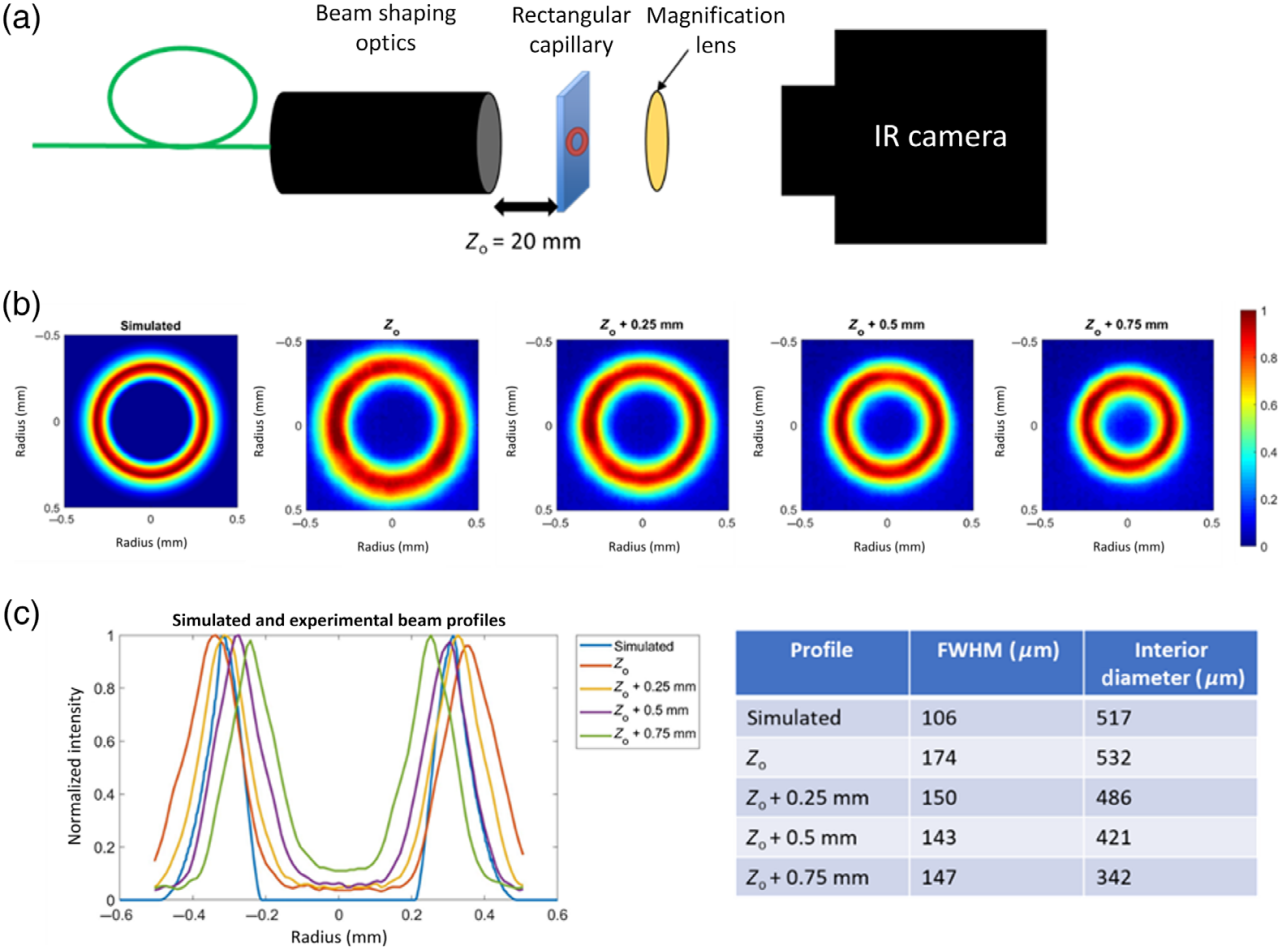
(a) Beam profiles were measured by irradiating a thin layer of water and imaging with an infrared camera. (b) and (c) Simulated and measured beam profiles show a uniform annular shape that decreases in diameter with increased distance from the beam-shaping optics.

Simulated and experimental beam profile measurements show an annular shape with good intensity uniformity around the circumference of the annulus [Fig. 5(b)]. The full width at half maximum (FWHM) width from outer to inner radius was larger for experimental vs. simulated beams [Fig. 5(c)]. The experimental FWHM outer beam diameter decreased from 874  μm (at position $Z_{o}=20\;\mathrm{mm}$ from the back side of focusing lens) to 795  μm (Z=20.25  mm) to 721  μm (Z=20.50  mm) to 643  μm (Z=20.75  mm) as the beam was measured in increments of 0.25 mm away from the focusing lens.

### 3.3 Computational Thermal Damage

**Fig. 6:**
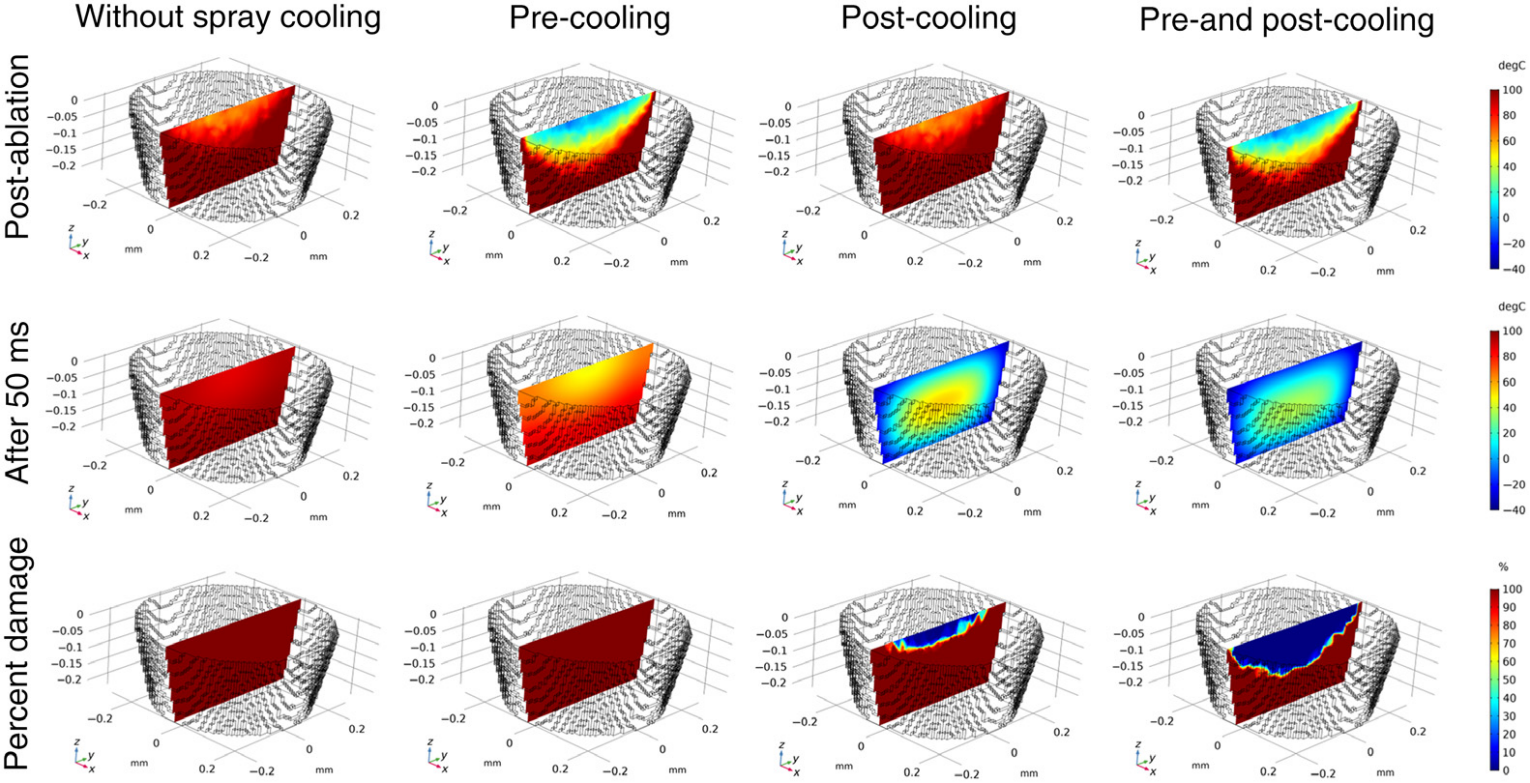
Temperature and thermal damage calculations for biopsies under different cooling schemes. Temperature profiles are shown immediately post ablation and after 50 ms of cooling time. Thermal damage profiles are shown as percentage of cell death.

**Fig. 7:**
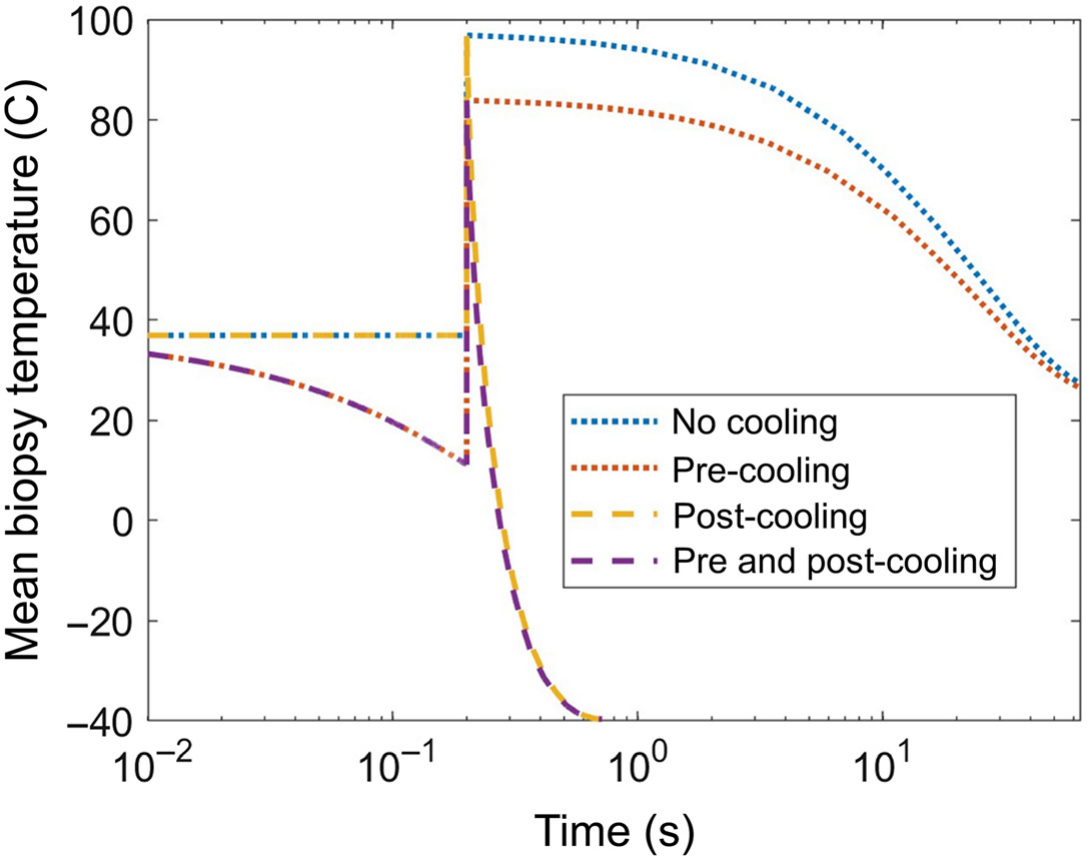
Average temperature within biopsy volume over time for each cooling scheme. The time axis is shown in log scale.

Computational results displaying biopsy volume geometry, post-ablation temperature, cooling profile, and resulting tissue damage are compared for biopsies with and without pre- and post-cooling are shown in Fig. 6. Computational results indicate that cooling does not significantly affect ablation depth. Results indicate that pre- and post-cooling works best in combination to preserve the largest volume of tissue. The model predicts that without post-cooling the entire biopsy would be thermally damaged at a level Ω>1 (Fig. 6, left two columns). With post-cooling alone a small fraction of tissue at the surface up to 55  μm into the tissue remains viable. With a combination of pre- and post-cooling a larger volume up to 140  μm into the tissue remains viable (Ω<1). The mean temperature in the simulated harvested microbiopsies over time, including before, during, and after the laser pulse is shown in Fig. 7. The time axis is shown on a log scale to display the orders of magnitude difference in cooling time for cases with and without post-cooling. From Figs. 6 and 7, we observe that pre-cooling limits the maximum temperature reached within the microbiopsy, while post-cooling with cryogen spray leads to much a much faster cooling time on the order of 50 ms. When used together, pre- and post-cooling minimize the extent of thermal damage.

**Fig. 8:**
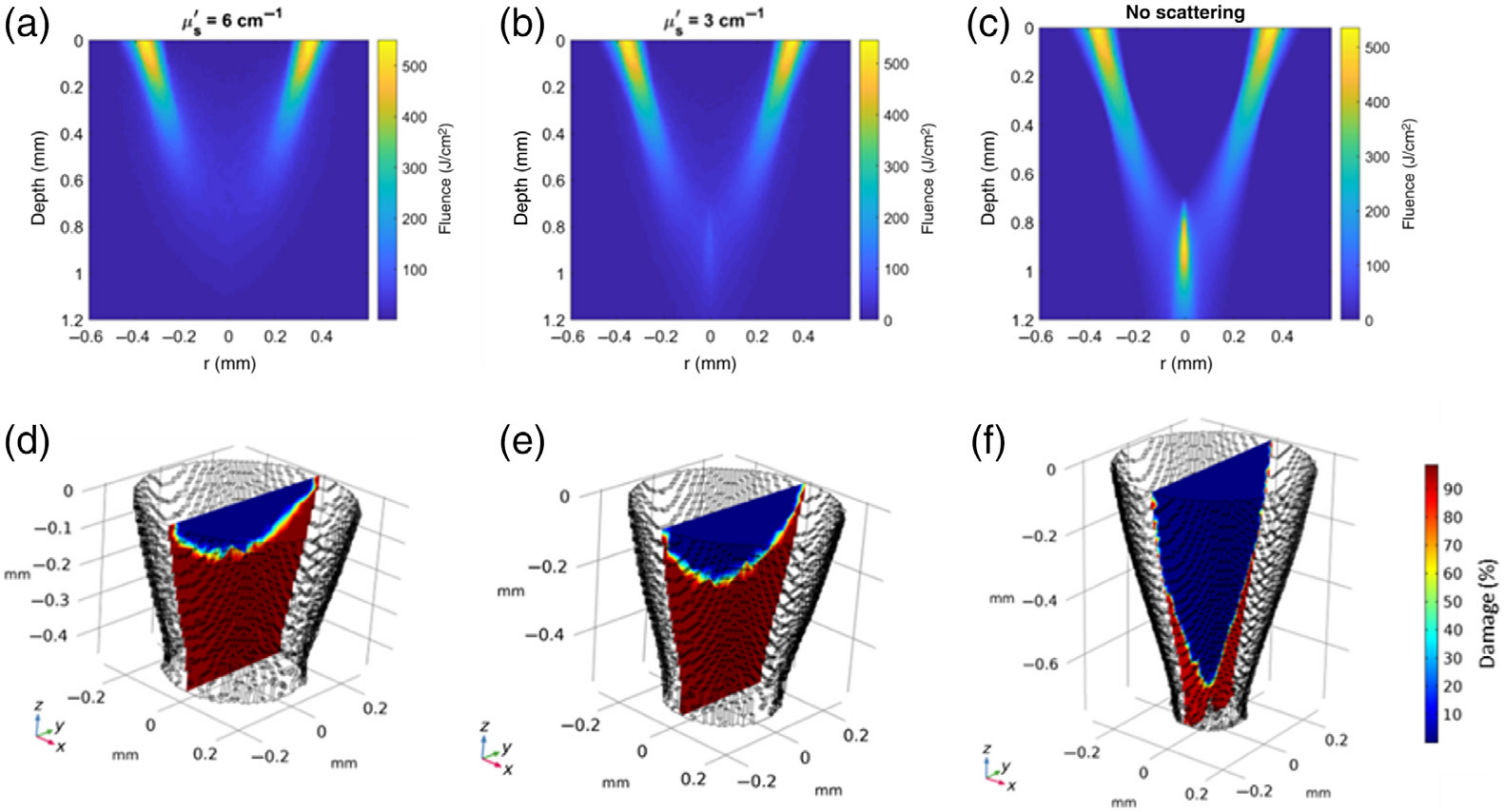
Computed fluence (top row) and tissue damage percentage (bottom row) of microbiopsies collected with pre- and post-cooling with different tissue scattering properties. (a) Normal scattering, ${\mu_{s}}^{\prime }=6\;{\mathrm{cm}}^{-1}$, (b) half scattering, ${\mu_{s}}^{\prime }=3\;{\mathrm{cm}}^{-1}$, and (c) no scattering.

The model also indicates that laser biopsy thermal damage is primarily due to light scattered from the annular beam into the center causing increased absorption, heating, and damage of the tissue. Results for normal scattering (${\mu_{s}}^{\prime }=6\;{\mathrm{cm}}^{-1}$), half scattering (${\mu_{s}}^{\prime }=3\;{\mathrm{cm}}^{-1}$), and zero scattering are compared with all cases including pre- and post-cooling (Fig. 8). By reducing scattering strength, depth of viable tissue (Ω<1) increased from 140  μm for normal scattering to 260  μm for half scattering, and 620  μm for zero scattering, suggesting that optical clearing methods that reduce tissue scattering could reduce microbiopsy damage. Optical clearing methods that could be used *in vivo* include mechanical optical clearing devices^41^ or chemicals such as glycerol which provide reversable optical clearing.^42^

### 3.4 Microbiopsy Harvest and Ablation Volume Measurements

**Fig. 9:**
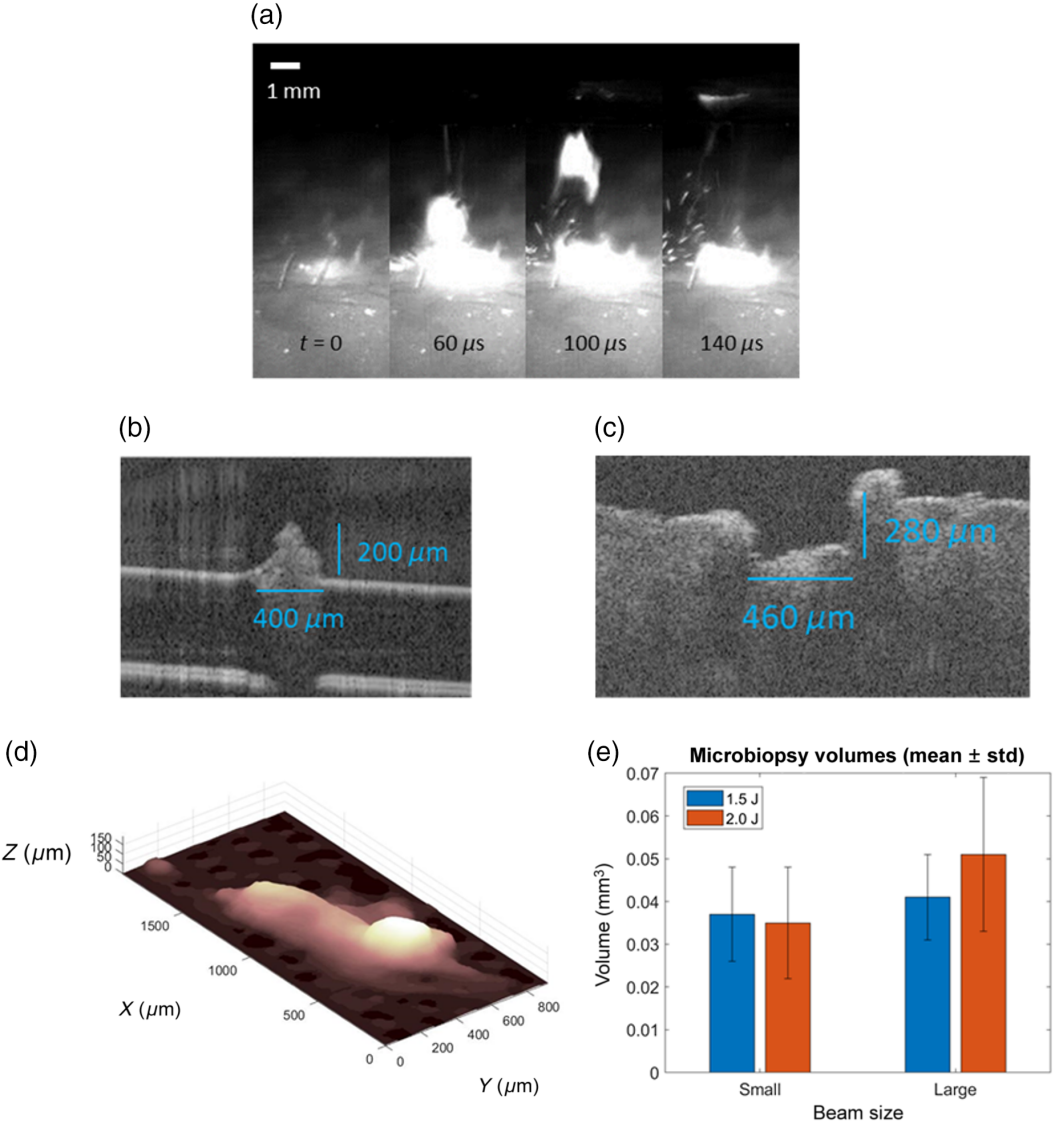
Biopsy harvest results: (a) select frames recorded by a fast camera (50,000  frames/s) of microbiopsy harvest. (b) OCT image of a harvested microbiopsy. (c) OCT image of a bulk tissue crater at the microbiopsy site. (d) 3D reconstruction of a microbiopsy volume. E) Mean microbiopsy volumes for two pulse energies and two beam diameters (estimated inner annulus diameters of 420 and 530  μm for small and large, respectively).

A pulse energy of 1.3 J was required to provide consistent and repeatable biopsy harvest when cooling was used. Fast camera videos show the microbiopsies ejecting from the center of the annular ablation region and reaching the coverslip after 100 to 150  μs [Fig. 9(a)]. OCT images show that the dimensions of ablation craters and harvested biopsies are on the order of 200 to 500  μm [Figs. 9(b) and 9(d)]. Ejected microbiopsy volumes collected on the glass slides increased with increasing beam diameter and volumes ranged from 0.02 to $0.08\;{\mathrm{mm}}^{3}$ [Fig. 9(e)].

### 3.5 Virtual H&E Images

**Fig. 10:**
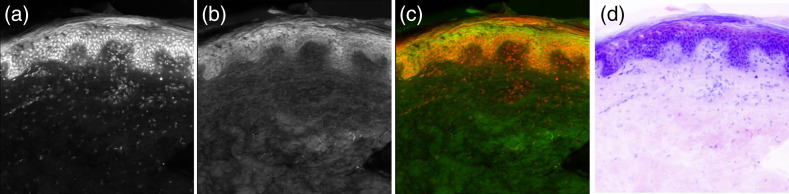
Virtual H&E imaging of bulk skin sample. (a) AO fluorescence; (b) SR101 fluorescence; (c) merged image; and (d) false colored virtual H&E image.

**Fig. 11:**
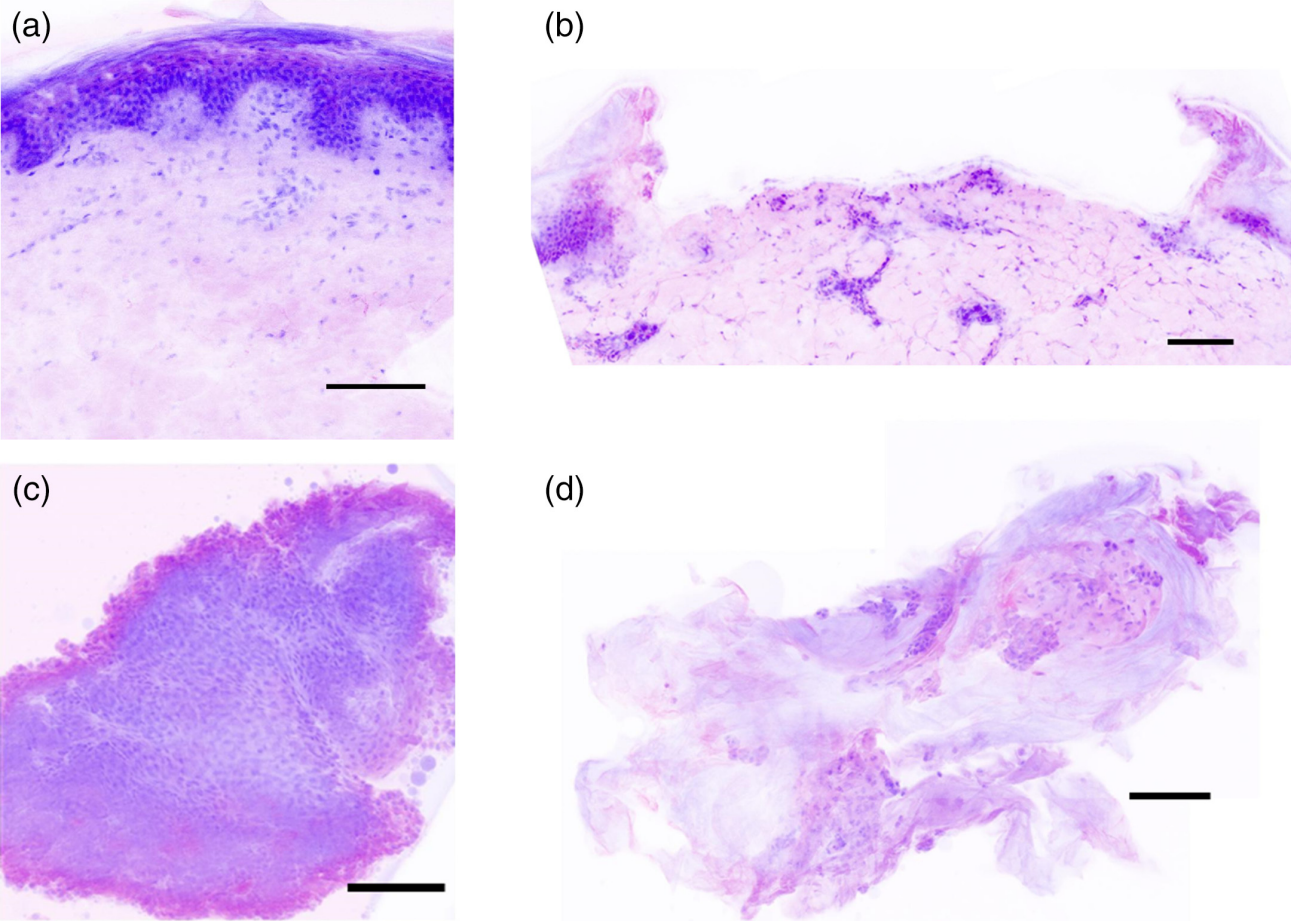
Examples of skin virtual H&E images, including (a) undamaged bulk skin, (b) microbiopsy crater in bulk skin, and (c) and (d) representative microbiopsies. Scale bars=100  μm.

**Fig. 12:**
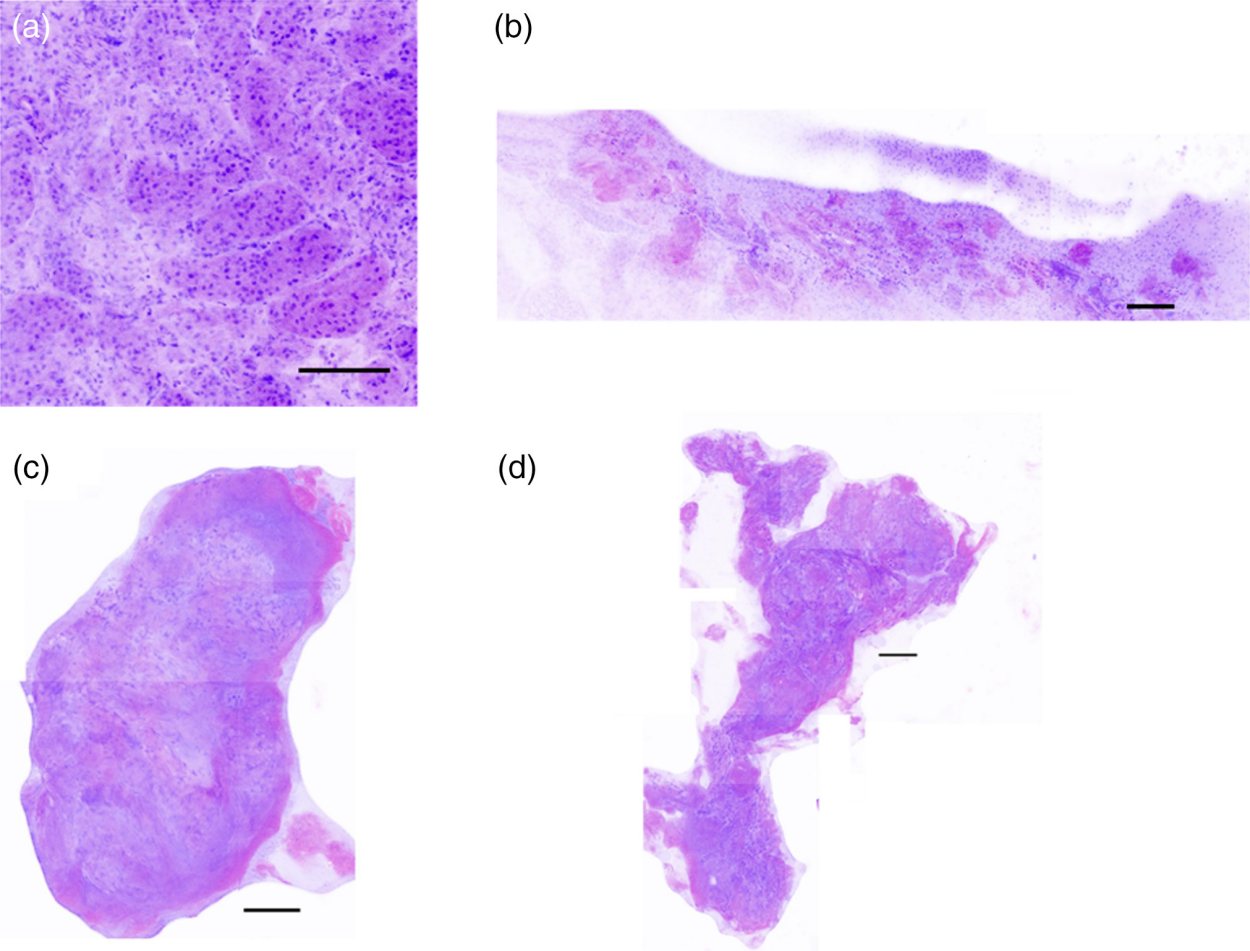
Example kidney virtual H&E images, including (a) undamaged bulk kidney, (b) microbiopsy crater in bulk kidney, and (c) and (d) representative microbiopsies. Scale bars=100  μm.

An example of the H&E virtual staining process for bulk porcine skin is shown in Fig. 10. AO fluorescence highlights the cell nuclei [Fig. 10(a)] while SR101 fluorescence highlights the cytosol and collagen [Fig. 10(b)]. The two channels were merged [Fig. 10(c)] and false colored to the purple and pink colors of H&E stains [Fig. 10(d)]. Example virtual H&E images for bulk porcine skin and kidney tissue, ablation craters, and harvested biopsies, are shown in Figs. 11 and 12, respectively.

The virtual H&E image of the skin ablation crater [Fig. 11(b)] suggests that the entire epidermis and a portion of the dermis was removed. The crater shows increased signal in the SR101 channel at the left and right edges of the crater in the epidermis, indicating thermal damage. No clear changes were observed in the image along the base of the crater in the dermis. While our model did not focus on predicting or preventing thermal damage to the bulk tissue near the microbiopsy crater, one might expect to see significant thermal damage along the full length of the crater due to high predicted temperature increase. The absence of observable damage along the base of the crater indicates less light scattering than predicted into the center of the annulus and/or limited thermal exposure due to pre- and post-cooling. Some skin microbiopsies appear to be entirely epidermis [Fig. 11(c)], while others appear to contain dermis and epidermis [Fig. 11(d)]. The biopsy edge in the epidermis shows increased signal in the SR101 channel [Fig. 11(c)].

The virtual H&E image of the kidney crater [Fig. 12(b)] shows increased signal in the SR101 channel along the full length of the ablation crater. Due to the weak mechanical properties of kidney, many smaller tissue fragments were ejected in addition to the larger microbiopsy. Some kidney microbiopsies largely retained their shape [Fig. 12(c)], while others were fragmented [Fig. 12(d)]. Portions of the biopsies appeared to have an increase in signal in the SR101 channel such as at the edges [Fig. 12(c)].

## 4 Discussion

Results of this study show that laser microbiopsy combined with virtual H&E imaging has potential to address several of the primary limitations of the traditional histopathology workflow. In contrast to traditional invasive biopsy tools which harvest tissues millimeters in size, laser microbiopsy harvests tissues that are several hundred microns in diameter with volumes $<0.1\;{\mathrm{mm}}^{3}$. We expect that this approach may increase the number of biopsies that can be tolerated by a patient in a single visit. One pathology where laser microbiopsy may provide value is for early detection of skin cancer. Clinicians are sometimes challenged to determine if a skin lesion is cancerous without biopsy. Twenty biopsies are taken per melanoma diagnosis and five biopsies per non-melanoma skin cancer diagnosis.^43^ Laser microbiopsy might be used, for example, as a first pass screening method to determine if a full-scale biopsy is needed. This could increase the number of lesions that could be tested and reduce the number of larger volume invasive biopsies required. The small scale of laser microbiopsy may also provide value intraoperatively to sample tissue near delicate tissue structures and preserve healthy tissue. Combination with a rapid analysis technique would provide feedback to the surgeon to aid in complete tumor resection.

In traditional invasive biopsy only a few 3- to 10-μm sections are analyzed out of a biopsy several millimeters in diameter. This equates to analyzing <1% of the harvested tissue volume. In contrast, the small scale of microbiopsy allows rapid imaging and viewing of the entire tissue volume.

Traditional tissue processing, sectioning, and staining require more than 20 min to days to complete and require a highly trained technician. In contrast, virtual H&E staining methods are very simple and require only ∼2  min to complete. No tissue pre-processing or sectioning is required. The tissue is placed into a single solution containing two stains for 2 min followed by a 20 s rinse step. Little time, expense, or training is required.

Microbiopsies were successfully harvested from both skin, which has a relatively high ultimate tensile strength and is considered a strong tissue,^44^ and kidney which has a relatively low ultimate tensile strength and is considered a mechanically weak tissue. Therefore, we expect laser microbiopsy to be achievable from various tissues with a range of mechanical properties.

The results of this study show compatibility of laser microbiopsy with virtual H&E imaging using confocal microscopy. Confocal microscopy provides similar H&E image quality to multiphoton microscopy at a much lower cost.^45^ Histological features including cell nuclei and collogen were observed in virtual H&E stained microbiopsies. A combination of some extent of thermal and mechanical damage was observed in harvested microbiopsies and bulk tissue harvest sites. Future work will continue to optimize the approach to further reduce this damage. An increase in SR101 signal was observed in areas of suspected thermal damage in the skin epidermis and kidney, suggesting that increase in SR101 accumulation is a good indicator of thermal damage in these structures. This increase in SR101 signal was not observed in the dermis. The observation suggests that either collagen in the epidermis was not thermally damaged, or that thermal damage occurred and did not lead to an observable change in SR101 accumulation.

While this study focused on virtual H&E as a diagnostic method, laser microbiopsy could potentially be used with a range of analytical techniques. For example, laser microbiopsy could be used with genetic or protein testing methods for analysis of cancer biomarkers. For example, laser microbiopsy may be valuable in applications such as determining BRAF mutation status for melanoma.^46^ DNA and RNA are expected to remain stable even at high temperatures for a short time.^47^^,^^48^ Therefore, we expect DNA/RNA testing to be compatible even with thermally damaged microbiopsies possibly including those harvested without spray cooling.

Harvested microbiopsies showed signs of mechanical damage in addition to the minor thermal injury. The ablation process that causes microbiopsy is explosive causing rapid ejection of the microbiopsy. The microbiopsy is ejected from the bulk tissue ∼60  μs after the beginning of the laser pulse and reaches the coverslip ∼80  μs after ejection. The 1 J short pulse setting used for these experiments has a mean full width half max pulse duration of 71  μs with a tail in the temporal profile that extends to 250  μs. Temporal profile measurements show that 56% of the pulse energy is delivered in the first 60  μs on average. Therefore, up to 44% of the pulse energy may be delivered after tissue ejection leading to unnecessary tissue heating. The microbiopsies can spin and fold as they travel in air to the coverslip. The collision with the coverslip can cause further mechanical damage. Therefore, future work will include developing a more controlled microbiopsy harvest to limit mechanical damage.

The current optical design of the laser microbiopsy device utilizes many free space optics, e.g., lenses that are 25 mm in diameter, which limits microbiopsy use to applications where sufficient space is available, such as for the skin or during open surgery. We aim to develop a miniaturized device with fiber optic delivery to allow implementation with endoscopic systems.

The Ho:YAG laser source used in this study is a commercial laser system that is cleared by the FDA for clinical use for indications including biopsy. The source is a class 4 laser that has potential to cause damage to the eye or skin. This risk is mitigated through precautions including the use of laser safety glasses during experimental procedures. Therefore, we expect laser microbiopsy can be implemented safely in a clinical setting when proper safety precautions are followed.

## 5 Conclusions

Laser microbiopsy with virtual H&E imaging shows promise as a potential rapid-minimally invasive tool for biopsy and intraoperative diagnosis. Microbiopsies were successfully harvested from both mechanically strong and weak tissues. A combination of pre- and post-cooling was required to preserve tissue integrity. Histological features including cell nuclei and collagen were observed in harvested microbiopsies.

## Code, Data, and Materials Availability

Code is available upon reasonable request.

## References

[1] Novis D. A. Zarbo R. J., “Interinstitutional comparison of frozen section turnaround time. A College of American Pathologists Q-Probes study of 32868 frozen sections in 700 hospitals,” Arch. Pathol. Lab. Med. 121(6), 559–567 (1997). APLMAS 0003-9985 9199619

[2] “Histopathology is ripe for automation,” Nat. Biomed. Eng. 1, 925 (2017). 10.1038/s41551-017-0179-5 31015707

[3] Zhang J. et al., “Nondestructive tissue analysis for ex vivo and in vivo cancer diagnosis using a handheld mass spectrometry system,” Sci. Transl. Med. 9(406), eaan3968 (2017). STMCBQ 1946-6234 10.1126/scitranslmed.aan3968 28878011 PMC5830136

[4] Sharma M. et al., “Design and characterization of a novel multimodal fiber-optic probe and spectroscopy system for skin cancer applications,” Rev. Sci. Instrum. 85(8), 1–10 (2014). RSINAK 0034-6748 10.1063/1.4890199 PMC4137875 25173240

[5] Gerami P. et al., “Development of a novel noninvasive adhesive patch test for the evaluation of pigmented lesions of the skin,” J. Am. Acad. Dermatol. 71(2), 237–244 (2014). JAADDB 0190-9622 10.1016/j.jaad.2014.04.042 24906614

[6] Park S. et al., “A novel microactuator for microbiopsy in capsular endoscopes,” J. Micromech. Microeng. 18(2), 025032 (2008). 10.1088/0960-1317/18/2/025032

[r7] Byun S. et al., “Barbed micro-spikes for micro-scale biopsy,” J. Micromech. Microeng. 15(6), 1279–1284 (2005). 10.1088/0960-1317/15/6/020

[r8] Gultepe E. et al., “Biopsy with thermally-responsive untethered microtools,” Adv. Mater. 25(4), 514–519 (2013). ADVMEW 0935-9648 10.1002/adma.201203348 23047708 PMC3832625

[9] Lin L. L. et al., “Microbiopsy engineered for minimally invasive and suture-free sub-millimetre skin sampling,” F1000Research 2, 120 (2013). 10.12688/f1000research.2-120.v2 24627782 PMC3907159

[10] Schütze K. Lahr G., “Identification of expressed genes by laser-mediated manipulation of single cells,” Nat. Biotechnol. 16(8), 737–742 (1998). 10.1038/nbt0898-737 9702771

[r11] Thalhammer S. et al., “Laser microtools in cell biology and molecular medicine,” Laser Phys. 13(5), 681–691 (2003).

[r12] Elvers D. et al., “Laser microdissection of biological tissues: Process optimization,” Appl. Phys. A Mater. Sci. Process. 80(1), 55–59 (2005). 10.1007/s00339-004-3017-z

[r13] Vogel A. et al., “Mechanisms of laser-induced dissection and transport of histologic specimens,” Biophys. J. 93(12), 4481–4500 (2007). 10.1529/biophysj.106.102277 17766336 PMC2098740

[14] Horneffer V. Linz N. Vogel A., “Principles of laser-induced separation and transport of living cells,” J. Biomed. Opt. 12(5), 054016 (2007). 10.1117/1.2799194 17994904

[15] Gareau D. S., “Feasibility of digitally stained multimodal confocal mosaics to simulate histopathology,” J. Biomed. Opt. 14(3), 034050 (2009). JBOPFO 1083-3668 10.1117/1.3149853 19566342 PMC2929174

[16] Gareau D. et al., “Tri-modal confocal mosaics detect residual invasive squamous cell carcinoma in Mohs surgical excisions Tri-modal confocal mosaics detect residual invasive squamous cell carcinoma in Mohs surgical excisions,” J. Biomed. Opt. 17(6), 066018 (2012). JBOPFO 1083-3668 10.1117/1.JBO.17.6.066018 22734774 PMC3381035

[17] Cahill L. C. et al., “Rapid virtual hematoxylin and eosin histology of breast tissue specimens using a compact fluorescence nonlinear microscope,” Lab. Investig. 98(1), 150–160 (2018). LAINAW 0023-6837 10.1038/labinvest.2017.116 29131161 PMC5752596

[r18] Kolb J. P. et al., “Virtual H & E histology by fiber-based picosecond two-photon microscopy,” Proc. SPIE 10882, 108822F (2019). PSISDG 0277-786X

[19] Giacomelli M. G. et al., “Virtual hematoxylin and eosin transillumination microscopy using epi-fluorescence imaging,” PLoS One 11(8), e0159337 (2016). POLNCL 1932-6203 10.1371/journal.pone.0159337 27500636 PMC4976978

[20] Glaser A. K. et al., “Light-sheet microscopy for slide-free non-destructive pathology of large clinical specimens,” Nat. Biomed. Eng. 1(7), 0084 (2017). 10.1038/s41551-017-0084 29750130 PMC5940348

[21] Fereidouni F. et al., “Microscopy with ultraviolet surface excitation for rapid slide-free histology,” Nat. Biomed. Eng. 1(12), 957–966 (2017). 10.1038/s41551-017-0165-y 31015706 PMC6223324

[22] Elfer K. N. et al., “DRAQ5 and eosin (‘D&E’) as an analog to hematoxylin and eosin for rapid fluorescence histology of fresh tissues,” PLoS One 11(10), e0165530 (2016). POLNCL 1932-6203 10.1371/journal.pone.0165530 27788264 PMC5082869

[23] Lin S.-E. et al., “Rapid pseudo-H&E imaging using a fluorescence-inbuilt optical coherence microscopic imaging system,” Biomed. Opt. Express 12(8), 5139–5158 (2021). BOEICL 2156-7085 10.1364/BOE.431586 34513247 PMC8407814

[24] Orringer D. A. et al., “Rapid intraoperative histology of unprocessed surgical specimens via fibre-laser-based stimulated Raman scattering microscopy,” Nat. Biomed. Eng. 1(2), 1–25 (2017). 10.1038/s41551-016-0027 PMC5612414 28955599

[25] Hollon T. C. et al., “Near real-time intraoperative brain tumor diagnosis using stimulated Raman histology and deep neural networks,” Nat. Med. 26, 52–58 (2020). 10.1093/neuros/nyz310_634 31907460 PMC6960329

[26] King J. B. et al., “Characterization of laser microbiopsy for sub-microliter tissue sampling,” Proc. SPIE 11631, 1163111 (2021). PSISDG 0277-786X 10.1117/12.2577258

[27] King J. B. et al., “Laser microbiopsy with virtual H&E imaging for rapid minimally invasive diagnosis,” Proc. SPIE 11949, 1194905 (2022). PSISDG 0277-786X 10.1117/12.2608931

[28] Latham W. P., “Characteristic analysis of a refractive axicon system for optical trepanning,” Opt. Eng. 45(9), 094302 (2006). 10.1117/1.2353119

[29] Vogel A. Venugopalan V., “Mechanisms of pulsed laser ablation of biological tissues,” Chem. Rev. 103(2), 577–644 (2003). CHREAY 0009-2665 10.1021/cr010379n 12580643

[30] Henyey L. G. Greenstein J. L., “Diffuse radiation in the galaxy,” Astrophys. J. 93, 70–83 (1941). ASJOAB 0004-637X 10.1086/144246

[31] Dai T. et al., “Comparative study of cryogen spray cooling with R-134a and R-404a: implications for laser treatment of dark human skin,” J. Biomed. Opt. 11(4), 041116 (2006). JBOPFO 1083-3668 10.1117/1.2338001 16965144

[32] Xu F. Seffen K. A. Lu T. J., “Temperature-dependent mechanical behaviors of skin tissue,” Int. J. Comput. Sci. 35(1), 92–101 (2008). 10.1002/9780470382776.ch1

[33] King J. B. et al., “Mechanisms of pulse modulated holmium:YAG lithotripsy,” J. Endourol. 35(S3), S29–S36 (2021). 10.1089/end.2021.0742 34910606 PMC8819872

[34] Welch A. J. et al., “Laser thermal ablation,” Photochem. Photobiol. 53(6), 815–823 (1991). PHCBAP 0031-8655 10.1111/j.1751-1097.1991.tb09896.x 1886940

[35] Pearce J., “Mathematical models of laser-induced tissue thermal damage,” Int. J. Hyperth. 27(8), 741–750 (2011). IJHYEQ 0265-6736 10.3109/02656736.2011.580822 22098359

[36] Katta N. et al., “Optical coherence tomography image-guided smart laser knife for surgery,” Lasers Surg. Med. 50, 202–212 (2018). LSMEDI 0196-8092 10.1002/lsm.22705 28782115

[37] Anvari B. et al., “Selective cooling of biological tissues: application for thermally mediated therapeutic procedures,” Phys. Med. Biol. 40(2), 241–252 (1995). PHMBA7 0031-9155 10.1088/0031-9155/40/2/003 7708851

[r38] Chen B. et al., “Theoretical study of cryogen spray cooling with R134a, R404A and R1234yf: comparison and clinical potential application,” Appl. Therm. Eng. 148(Aug. 2018), 1058–1067 (2019). ATENFT 1359-4311 10.1016/j.applthermaleng.2018.11.117

[39] Xin H. et al., “Histopathological evaluation of the R134a multipulsed spray cooling assisted 1210 nm laser lipolysis by the murine model in vivo,” Lasers Surg. Med., 1–11 (2022). LSMEDI 0196-8092 10.1002/lsm.23607 36229977

[40] Jacques S. L., “Optical properties of biological tissues: a review,” Phys. Med. Biol. 58(14), 5007–5008 (2013). PHMBA7 0031-9155 10.1088/0031-9155/58/14/5007 23666068

[41] Rylander C. G. et al., “Mechanical tissue optical clearing devices: enhancement of light penetration in ex vivo porcine skin and adipose tissue,” Lasers Surg. Med. 40(10), 688–694 (2008). LSMEDI 0196-8092 10.1002/lsm.20718 19065559 PMC2605205

[42] Weny X. et al., “In vivo skin optical clearing by glycerol solutions: mechanism,” J. Biophotonics 3(1–2), 44–52 (2010). 10.1002/jbio.200910080 19937846

[43] Privalle A. et al., “Number of skin biopsies needed per malignancy: comparing the use of skin biopsies among dermatologists and nondermatologist clinicians,” J. Am. Acad. Dermatol. 82(1), 110–116 (2020). JAADDB 0190-9622 10.1016/j.jaad.2019.08.012 31408683

[44] Walsh J. T. Deutsch T. F. Walsh J. T., “Pulsed CO2 laser ablation of tissue: effect of mechanical properties,” IEEE Trans. Biomed. Eng. 36(12), 1195–1201 (1989). IEBEAX 0018-9294 10.1109/10.42114 2606495

[45] Yoshitake T. et al., “Direct comparison between confocal and multiphoton microscopy for rapid histopathological evaluation of unfixed human breast tissue,” J. Biomed. Opt. 21(12), 126021 (2016). JBOPFO 1083-3668 10.1117/1.JBO.21.12.126021 28032121 PMC5197052

[46] Cheng L. et al., “Molecular testing for BRAF mutations to inform melanoma treatment decisions: a move toward precision medicine,” Mod. Pathol. 31(1), 24–38 (2018). MODPEO 0893-3952 10.1038/modpathol.2017.104 29148538 PMC5758899

[47] Karni M. et al., “Thermal degradation of DNA,” DNA Cell Biol. 32(6), 298–301 (2013). 10.1089/dna.2013.2056 23621849

[48] Fabre A. L. et al., “An efficient method for long-term room temperature storage of RNA,” Eur. J. Hum. Genet. 22(3), 379–385 (2014). EJHGEU 1018-4813 10.1038/ejhg.2013.145 23860045 PMC3925273

