# Tissue harvest with a laser microbiopsy (Erratum)

**DOI:** 10.1117/1.JBO.28.2.029801

**Published:** 2023-02-28

**Authors:** Jason B. King, Nitesh Katta, Sapun H. Parekh, Thomas E. Milner, James W. Tunnell

**Affiliations:** a The University of Texas at Austin, Department of Biomedical Engineering, Austin, Texas, United States; b University of California Irvine, Beckman Laser Institute and Medical Clinic, Irvine, California, United States

## Abstract

The erratum adds references to relevant prior work using lasers for microdissection of a tissue section.

This article [*J. Biomed. Opt.* **27**(12), 125001 (2022) doi: https://doi.org/10.1117/1.JBO.27.12.125001 was originally published on 14 December 2022 without references to some relevant prior work using lasers for microdissection of a tissue section.

The following text and references were added to the 2nd paragraph of the Introduction section:

While lasers have been utilized previously for laser microdissection to harvest cells from thin histologic sections or cell cultures,^10^^–^^14^ the laser microbiopsy approach described here harvests three-dimensional tissue volumes from thick tissues. In addition, we describe a photothermal cutting mechanism as opposed to plasma mediated ablation used for laser microdissection.^13^^,^^14^

The article was corrected and republished on 14 February 2023.
